# RNA-seq Analysis Reveals Potential Synergic Effects of Acetate and Cold Exposure on Interscapular Brown Adipose Tissue in Mice

**DOI:** 10.3390/biology12101285

**Published:** 2023-09-26

**Authors:** Hongtao Ou, Qingyan Chen, Zhongjing Lin, Yang Yang, Peixin Wang, Natthida Sriboonvorakul, Shaoling Lin

**Affiliations:** 1 College of Food Science, Fujian Agriculture and Forestry University, Fuzhou 350002, China; hongtao.ou@fafu.edu.cn (H.O.);; 2 Shenzhen Research Institute, The Hong Kong Polytechnic University, Shenzhen 518051, China; 3 Department of Clinical Tropical Medicine, Faculty of Tropical Medicine, Mahidol University, Bangkok 10400, Thailand

**Keywords:** acetate, cold exposure, brown adipose tissue, RNA-seq analysis

## Abstract

**Simple Summary:**

Our study aims to understand the impact of acetate intervention at the systemic level before a short period of cold exposure on brown adipose tissue in mice. The results show that acetate may demonstrate a synergistic effect with cold exposure to induce obvious transcriptional changes in brown adipose tissue. These analyses provided novel evidence for understanding the regulatory effects of acetate on brown adipose tissue and highlighted its potential for obesity treatment.

**Abstract:**

Brown adipose tissue (BAT) exhibits remarkable morphological and functional plasticity in response to environmental (e.g., cold exposure) and nutrient (e.g., high-fat diet) stimuli. Notably, a number of studies have showed that acetate, the main fermentation product of dietary fiber in gut, profoundly influences the differentiation and activity of BAT. However, the potential synergic or antagonistic effects of acetate and cold exposure on BAT have not been well examined. In the present study, the C57BL/6J mice were treated with acetate at the systemic level before a short period of cold exposure. Physiological parameters including body weight, blood glucose, and Respiratory Exchange Ratio (RER) were monitored, and thermal imaging of body surface temperature was captured. Moreover, the transcriptome profiles of interscapular BAT were also determined and analyzed afterwards. The obtained results showed that acetate treatment prior to cold exposure could alter the gene expression profile, as evidenced by significant differential clusters between the two groups. GO analysis and KEGG analysis further identified differentially expressed genes being mainly enriched for a number of biological terms and pathways related to lipid metabolism and brown adipose activity such as “G-protein-coupled receptor activity”, “cAMP metabolic process”, “PPAR signaling pathway”, and “FoxO signaling pathway”. GSEA analysis further suggested that activation status of key pathways including “PPAR signaling pathway” and “TCA cycle” were altered upon acetate treatment. Taken together, our study identified the potential synergistic effect of acetic acid with cold exposure on BAT, which highlighted the positive dietary and therapeutic aspects of acetate.

## 1. Introduction

Obesity has emerged as a pressing public health concern, with 39% and 13% of adults being classified as overweight and obese, respectively. Moreover, obesity has been identified as a major contributor to the development of type 2 diabetes [1], dyslipidemia, hypertension, coronary heart disease, stroke, and many types of cancer.

Notably, major characteristics of obesity include hyperplasia and hypertrophy of adipocytes in white adipose tissue, which has the capacity to store excessive energy. Interestingly, unlike well-known white adipose tissue, there exists another type of adipose tissue in the human body known as brown adipose tissue, consisting mainly of brown adipocytes [2]. Brown adipocytes are distinguished by their unique uncoupling capacity mediated by mitochondrial brown fat uncoupling protein 1 (UCP-1). Increasing evidence has revealed that brown adipose tissue is the most efficient energy-dissipating tissue, which could produce 300 watts/kg of heat compared to 1 watt/kg in all other tissues [3]. Therefore, increasing brown adipose tissue mass and/or its activity is expected to be a promising strategy to relieve metabolic disorders and combat obesity [4]. Several transcriptional factors (and co-factors) and pathways such as PPARs, C/EBPs, PGC-1, and cAMP signaling have been identified as key regulators for activation and differentiation of brown adipocytes [5,6,7,8].

Short-chain fatty acids (SCFAs) are organic acids containing 1–6 carbon atoms, which are mainly produced by the fermentation of indigestible carbohydrates by colonic anaerobic bacteria, with acetic acid being the most abundant in circulation [9]. Studies already revealed that acetate plays important roles such as regulating energy homeostasis, controlling appetite, and improving obesity. However, there still lacks a universally accepted consensus on the regulatory roles of acetate on brown adipose tissue [10]. For example, a number of studies showed acetate intervention at the systemic level led to increased brown adipose tissue content and activity. Sahuri-Arisoylu et al. demonstrated that intraperitoneal injection of acetate could induce browning of the inguinal white adipose tissue and thus enhance energy expenditure in diet-induced obesity [11]. H Yamashita et al. also found that acetate may improve glucose tolerance and insulin resistance in mice via the AMPK pathway [12]. These results suggest that acetate may promote the differentiation of brown adipocytes and increase their thermogenic efficiency. However, there were certain studies that reported the opposite findings that acetate may reduce the thermogenic capacity of brown adipose tissue and inhibit the browning of adipose tissue. For example, Sun et al. reported local acetate administration at the interscapular brown adipose tissue using a pump implantation that reduced the activity of the brown adipose tissue and caused its whitening in C57B6 mice [13]. The same group also identified a subpopulation of adipocytes in brown adipose tissue that could hamper thermogenesis activity in a paracrine way by releasing acetate [14].

Notably, cold exposure has been identified as a well-studied means to activate brown adipose tissue and stimulate its glucose and lipid uptake through mitochondrial uncoupling [15,16,17]. The underlying mechanisms mainly involve the activation of the sympathetic nervous system upon cold exposure, which activates the β-adrenergic receptor [18] and stimulates the cAMP-dependent signaling pathways in brown adipocytes [19]. However, the potential synergic or antagonistic effects of acetate and cold exposure (a well-known physiological stimulus) on brown adipose tissue have not been well examined. Therefore, in the present study, we determined the regulatory effects of acetate administration before a short period of cold exposure on the interscapular brown adipose tissue in C57BL/6J mice.

## 2. Materials and Methods

### 2.1. Materials and Reagents

C57BL/6J mice and rodent diet with 10% kcal% fat (ND diet) was purchased from Wu experiment Animal Co., Ltd. (Fuzhou, China). Sodium acetate was purchased from Pharmaceutical Group Chemical Reagent Co., Ltd. (Shanghai, China). QIAzol Lysis Reagent was purchased from QIAGEN (Hilden, Germany). Phosphate-buffered saline (PBS) and normal saline were obtained from Servicebio (Wuhan, China). Hydrogen peroxide (3%) was bought from the China National Pharmaceutical Group Chemical Reagent Co., Ltd. (Shanghai, China).

### 2.2. Animals

**Figure 1:**
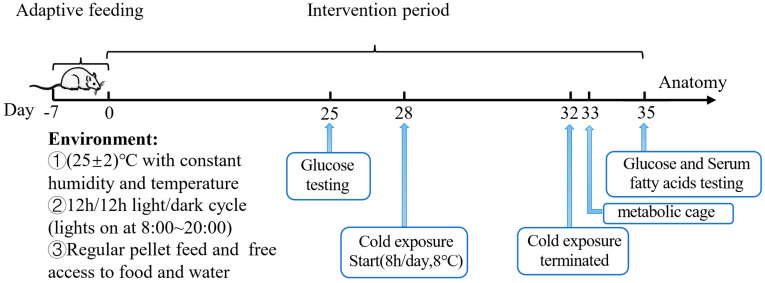
Animal experiments design.

After 7 days of acclimation, 5-week-old male C57BL/6J mice were divided into two groups with 5 mice in each group and received the following intervention for 35 days: (1) Cold Exposure (CE) group: mice with free access to normal drinking water and treated with cold exposure (8 °C) for 5 days (Day 28–32, 8 h daily, 12:00–20:00); (2) Acetate_Per Oral+Cold Exposure (Ace_po+CE) group: mice with free access to drinking water containing 5% acetate and treated with cold exposure (8 °C) for 5 days (Day 28–32, 8 h daily, 12:00–20:00) (Figure 1). All mice were housed with a 12 h/12 h light/dark cycle (lights on at 8:00~20:00) and free access to food. During the feeding period, the body weight, food intake, and water intake of mice were monitored weekly.

### 2.3. Metabolic Caging Analysis

On day 33, the mice were transferred into the metabolic cages for a 22 h metabolic experiment (14:15~12:15) to measure O_2_ consumption and CO_2_ production in individual mice and calculate the corresponding Respiratory Exchange Ratio (RER) to reflect energy expenditure.

### 2.4. Oral Glucose Tolerance Test (OGTT)

All mice were fasted overnight before blood was taken from the tail-end vein. During OGTT, the fasting blood glucose of the mice was measured as the starting value (0 min), and then a 10% glucose solution was administered orally. The blood glucose values of the mice were recorded at 15 min, 30 min, 60 min, and 120 min. Glucose was measured by a OneTouch Ultra blood glucose meter (Sinocare Inc., Changsha, China).

### 2.5. Measurement of Serum Lipid Profiles

Blood was collected by eyeball removal before cervical dislocation. After resting for 30 min, the serum was separated by centrifugation at 3000 rpm for 10 min. The obtained serum was used to measure total triglycerides (TG), total cholesterol (TCHO), high-density lipoprotein cholesterol (HDL-C), and low-density lipoprotein cholesterol (LDL-C) using a Hitachi 7600-110 automatic biochemical analyzer (Tokyo, Japan).

### 2.6. Dissection of Interscapular Brown Adipose Tissue (iBAT)

After CO_2_ anesthesia, mice were euthanized by cervical dislocation. The iBAT were dissected and washed with phosphate-buffered saline (PBS) according to published guideline [20]. The tissues were transferred into cryogenic tubes and snap-frozen in liquid nitrogen, then stored at −80 °C for subsequent analysis.

### 2.7. Infrared Imaging

The mice were subjected to a 3 h exposure to a low-temperature environment (8 °C), during which the mice had unrestricted access to food and water. After the exposure, the mice were placed on a flat surface, and the thermal imaging of body surface temperature was captured by a FLIR ONE Pro LT infrared thermal imaging camera (Teledyne FLIR LLC, Wilsonville, OR, USA) and analyzed by FLIR Tools 2.2.2 software (Teledyne FLIR LLC, Wilsonville, OR, USA).

### 2.8. RNA-Seq

The total RNA was extracted from adipose tissue using QIAzol Lysis Reagent. The integrity of the RNA was confirmed using a Bioanalyzer 2100 (Agilent Technologies, Santa Clara, CA, USA). Then, the cDNA library was constructed according to the procedures of TruSeq RNA Library Prep Kit (Illumina, San Diego, CA, USA). RNA-seq was performed on the Illumina HiSeq 1500 platform. For statistical analysis of differentially expressed genes (DEGs), TPM was used for data normalization, the BH (FDR) method was applied for multiple testing correction, and the DESeq2 was used for calculations. Genes that had a corrected *p*-value < 0.05 and |log_2_FC| > 1 were considered as DEGs. A volcano plot was used to visually display the distribution of the differential expression and *p* values of all statistically tested genes, and the plot was constructed with the log_2_FC values on the *x*-axis and −log10 (*p*-value) on the *y*-axis. The TBtools software (v1.120) was used to perform gene clustering analysis and generate the heatmap of the DEGs. GO enrichment analysis was conducted using the GO database, and only pathways with a *p*-value < 0.05 were selected. KEGG Pathway enrichment analysis was performed by KOBAS. GSEA analysis was performed using GSEA4.0.

### 2.9. Statistical Analysis

The experimental data were presented as mean ± Standard Deviation (SD). Normality of the data was confirmed by the Kolmogorov–Smirnov test. The student’s *t* test or ANOVA with Tukey post hoc tests were used for statistical analysis between groups. *p* < 0.05 was considered as statistically significant. Microsoft Excel 2021 and Graphpad Prism 9 were used for statistical analysis and data graphing.

## 3. Results

### 3.1. The Effects of Acetate Treatment on Physiological and Metabolic Characteristics of Cold-Stimulated Mice

First, to confirm the cold-exposure murine model was established successfully, the elevation of characteristic BAT gene (Ucp1) was determined and compared with normal mice without cold exposure according to previous literature [21,22]. As expected, the characteristic BAT gene Ucp1 significantly upregulated during cold exposure (Supplementary Figure S1).

**Figure 2:**
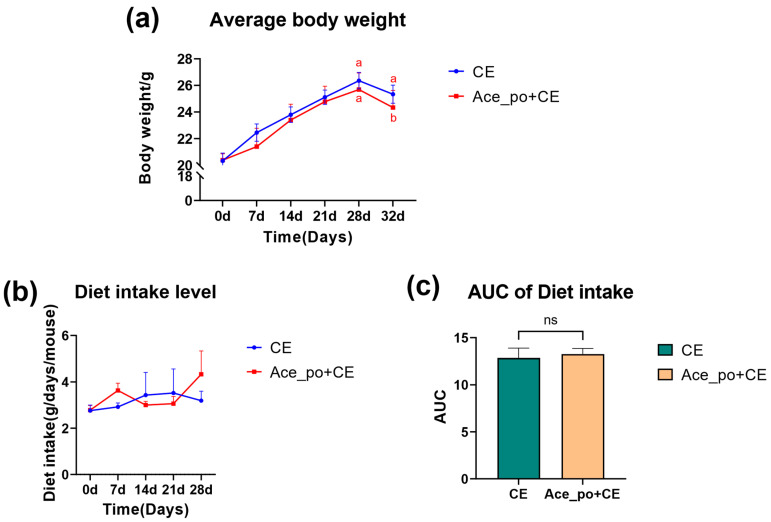
The effects of acetate pre-treatment before cold exposure (Day 0–35) on the body weight and diet intake. (**a**) Average body weight and (**b**) diet intake level of mice. (**c**) The AUC of Diet intake. All data are presented as mean ± SD. AUC: Area under the curve, ns: no significance. Different lowercase letters indicate significant differences among different treatments.

Next, as shown in Figure 2a, the treatment of acetate solution showed little impact on body weight gain since the growth curves of body weight in both groups were almost identical before cold exposure. Consistently, as shown in Figure 2b, no significant differences in food intake were observed between the two groups, indicating that the intervention of acetate showed little impact on the appetite of mice. Notably, after cold exposure, the mice body weight showed a significant decrease in Ace_po+CE group (*p* = 0.0229, b.w. at day 32 compared to b.w. at 28). Although a similar declining trend was also observed in the CE group at day 32, however, the decrease did not reach statistical significance compared to day 28 (*p* = 0.2110), suggesting the acetate pre-treatment may enhance energy expenditure during cold exposure, thus resulting in more obvious body weight loss.

**Figure 3:**
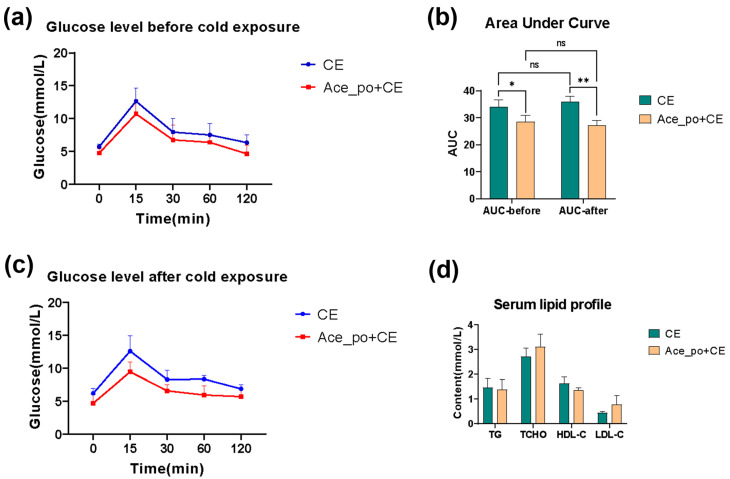
The effects of acetate pre-treatment on the oral glucose tolerance test (OGTT) and serum lipid profile. (**a**) The line chart of OGTT test on day 25 (before cold exposure). (**b**) The line chart of OGTT test on day 35 (after cold exposure). (**c**) The AUC of OGTT test. (**d**) Changes in serum lipid profile [Triglyceride (TG), Total cholesterol (TCHO), High Density Lipoprotein-cholesterol (HDL-C), and Low-Density Lipoprotein-cholesterol (LDL-C)] between the CE and Ace_po+CE group. All data are presented as mean ± SD, * *p* < 0.05, ** *p* < 0.01. AUC: Area under the curve, ns: no significance.

Next, the potential impact of acetate treatment on blood glucose level was determined. As depicted in Figure 3a,b, the mice with acetate treatment exhibited a slight reduction in blood glucose levels after glucose challenge both before and after cold exposure. The analysis of the area under the curve (AUC) derived from the OGTT data also confirmed that the Ace_po+CE group had a significantly lower rise in blood glucose during the OGTT (*p*  <  0.05) when compared to CE group. In addition, the analysis also revealed that no significant difference was found between the AUC values determined on day 25 (before cold exposure) and on day 35 (after cold exposure) in both the control and treated groups (Figure 3c).

Notably, no significant difference in blood lipid levels was observed between these two groups as shown in Figure 3d. Considering the mice in the current experiments were fed with an ND diet, and all tested blood lipids were within the normal range [23], this might be the reason that the acetate treatment before cold exposure showed no obvious impact on the serum lipid profile.

**Figure 4:**
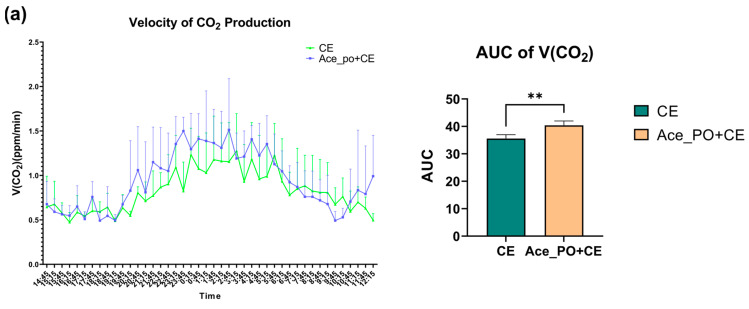

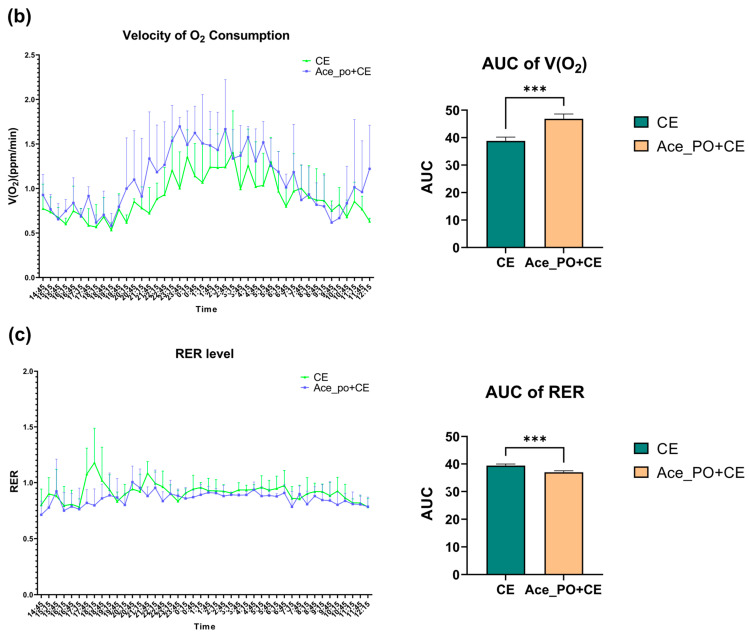
The effect of acetate pre-treatment before cold exposure on metabolic rate measured by respiration chamber on Day 33. (**a**) Velocity of carbon dioxide production and its AUC. (**b**) Velocity of oxygen consumption and its AUC. (**c**) RER level and its AUC. All data are presented as mean ± SD, ** *p* < 0.01, *** *p* < 0.001. AUC: Area under the curve; RER: respiratory exchange ratio.

To further explore the acetate treatment before cold exposure on the metabolic characteristics of mice, the carbon dioxide production, oxygen consumption, and corresponding Respiratory Exchange Ratio were measured by respiration chamber. As shown in Figure 4a,b, the mice with both acetate treatment and cold exposure showed a higher peak of V(CO2) and V(O2) during 19:15~5:45; moreover, the comparison of AUC shows that the rate of carbon dioxide production and oxygen consumption in the Ace_po+CE group are significantly higher than those in the CE group, indicating the mice in Ace_po+CE group had higher levels of carbon dioxide production and oxygen consumption (i.e., higher levels of total metabolic rate). Accordingly, the Respiratory Exchange Ratio (RER) level, which reflected the metabolic substrate used, showed that the Ace_po+CE group had a lower RER level than the CE group, suggesting more fat being used as metabolic substrates in the mice of the Ace_po+CE group. Taken together, the results indicate that acetate treatment before cold exposure enhanced the metabolic activity of mice, which might be attributed to the regulatory effects on BAT.

**Figure 5:**
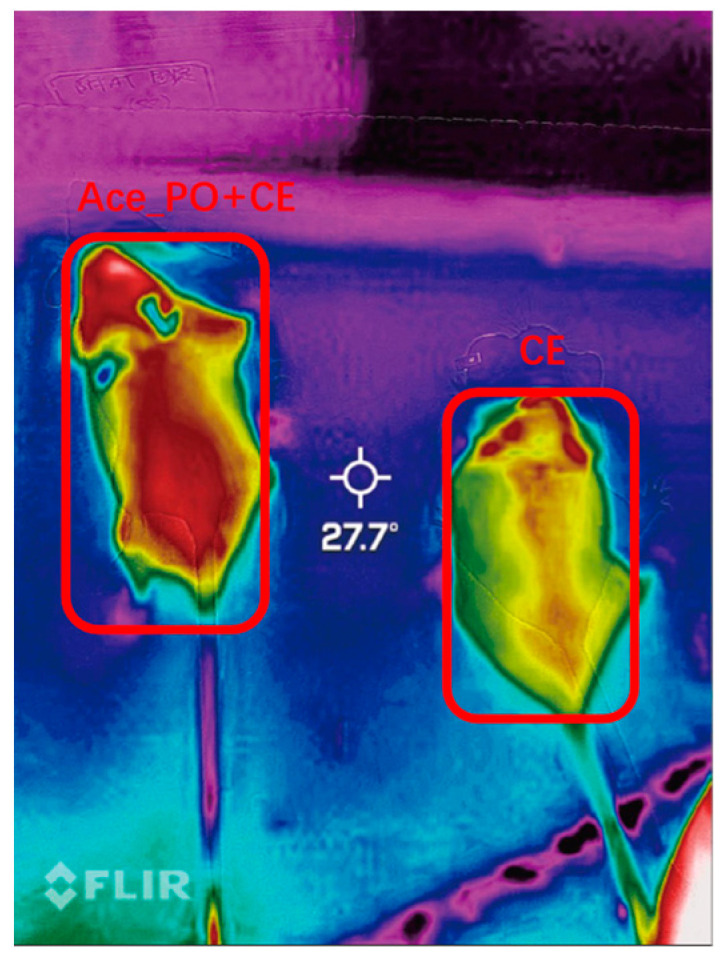
Thermal imaging of body surface temperature of mice in the Ace_po+CE group (**left**) and CE group (**right**).

To further determine the difference in thermogenesis ability between two groups, the thermal imaging of body surface temperature was captured. As shown in Figure 5, the body surface temperature of the Ace_po+CE group mice was higher than that of the CE group, also indicating a possible increase in thermogenesis ability upon treatment.

### 3.2. Transcripts Regulated by Acetate Treatment in Interscapular Brown Adipose Tissue

**Figure 6:**
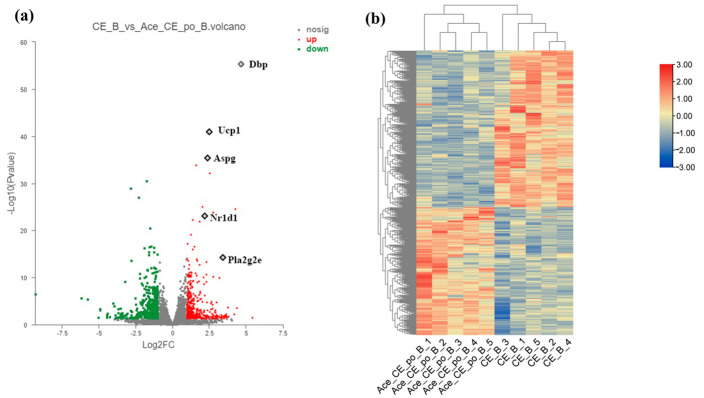
Differential Expression Genes (DEGs) of brown adipose tissue (BAT) samples from mice in the CE group and Ace_po+CE group. (**a**) The volcano plot of DEGs between CE group and Ace_po+CE group. (**b**) Cluster analysis of DEGs in each group of mice base on Deseq2. DEGs with significantly downregulation (padjust < 0.05 and $\log_{2}\mathrm{FC}>1$) was marked green; DEGs with significantly upregulation (padjust < 0.05 and $\log_{2}\mathrm{FC}<-1$) was marked red. Dbp: D site-binding protein; Ucp1: uncoupling protein 1; Aspg: asparaginase; Nr1d1: nuclear receptor subfamily 1, group D, member 1; Pla2g2e: phospholipase A2, group IIE.

As shown in the volcanic plot (Figure 6a), 485 differentially expressed genes (DEGs) (268 upregulated genes and 217 downregulated genes) were identified between iBAT samples isolated from the CE group and Ace_po+CE group based on the criteria of *p*_adjust_ < 0.05 and |log_2_FC| > 1. It is worth noting that several key genes involved in adipocyte differentiation and metabolism were identified as DEGs, including Ucp1 (|log_2_FC| = 2.5), Pla2g2e (|log_2_FC| = 3.4), Dbp (|log_2_FC| = 4.7), Aspg (|log_2_FC| = 2.4), and Nr1d (|log_2_FC| = 2.2). Furthermore, cluster analysis of DEGs also clearly presented the distinct gene expression profiling existing between two groups (Figure 6b), showing the obtained gene expression profiles were highly divergent between two groups. Taken together, these results clearly indicate that the acetate treatment may demonstrated a synergistic effect with cold exposure to regulate the iBAT functions via influencing the gene expression profile at the transcriptional level.

### 3.3. Gene Ontology Analysis and KEGG Enrichment of the Differentially Expressed Genes

**Figure 7:**
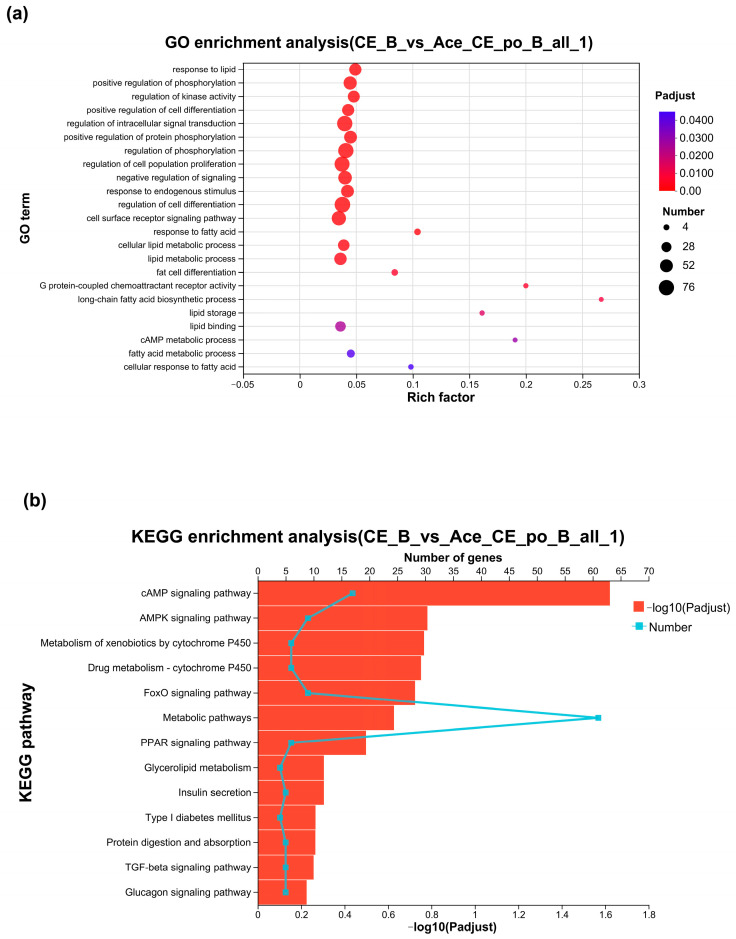
(**a**) Gene Ontology Analysis of DEGs between the CE group and Ace_po+CE group. (**b**) The bar chart of KEGG enrichment analysis of DEGs between the CE group and Ace_po+CE group. KEGG: Kyoto Encyclopedia of Genes and Genomes.

Next, the identified DEGs were subjected to Gene Ontology (GO) analysis for functional annotation. As shown in Figure 7a, the obtained results showed that among the 491 significantly enriched GO terms, 23 GO terms were found to be related to lipid metabolism and brown adipose activity. Among these terms, the “G-protein-coupled receptor activity pathway and the lipid binding pathway” were classified as molecular function (MF) pathways, while the remaining 21 pathways were biological process (BP) pathways. Particularly, “long-chain fatty acid biosynthetic process”, “G-protein-coupled receptor activity”, “cAMP metabolic process”, “lipid storage”, “response to fatty acid”, and “fat cell differentiation” showed Rich factor > 0.1, suggesting that acetate may perform its function mainly through these pathways. Only the G-protein-coupled receptor activity pathway and the lipid binding pathway were classified as molecular function (MF) pathways, while the remaining 21 pathways were biological process (BP) pathways.

The KEGG pathway enrichment was next performed using KOBAS. As shown in Figure 7b, between the CE group and Ace_po+CE groups, a total of 279 pathways were enriched under the condition of *p* < 0.05, of which 13 pathways were related to lipid metabolism, iBAT activity, and iBAT differentiation, including the classical pathways like the “cAMP signaling pathway”, “FoxO signaling pathway”, and “PPAR signaling pathway”, etc.

### 3.4. Gene Set Enrichment Analysis of the Differentially Expressed Genes

**Figure 8:**
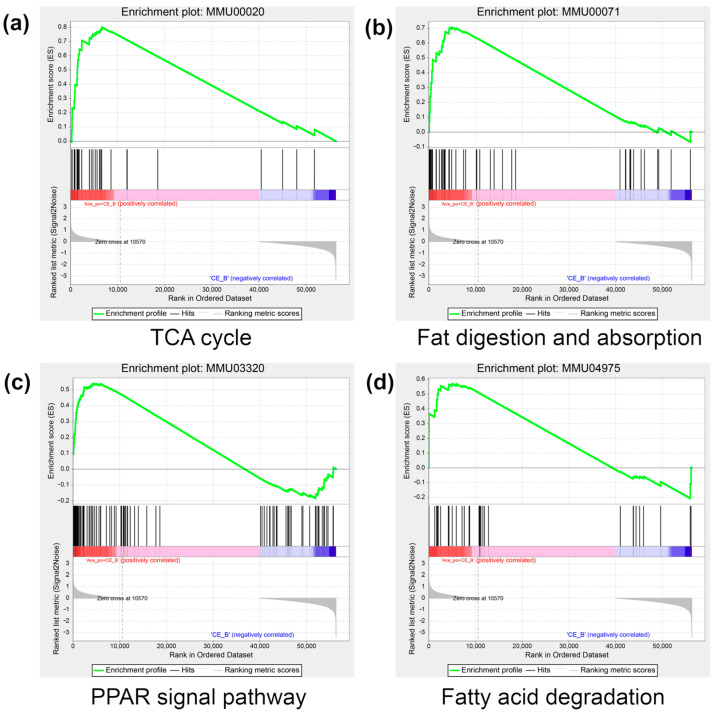
The result of GSEA in the Ace_po+CE group. (**a**) The enrichment result of the TCA cycle. (**b**) The enrichment result of fat digestion and absorption. (**c**) The enrichment result of the PPAR signal pathway. (**d**) The enrichment result of fatty acid degradation. The colors (red to blue) represent the decreasing trend of log_2_FC of genes. GSEA: Gene Set Enrichment Analysis; TCA cycle: tricarboxylic acid cycle; PPAR: Peroxisome proliferator-activated receptor.

To further gain biological insight regarding the DEGs, gene set enrichment analysis (GSEA) was also performed on the DEGs. As shown in Figure 8a–d, the analysis revealed that four pathways closely associated with lipid metabolism and brown adipose activity were significantly upregulated, including the Tricarboxylic acid (TCA) cycle digestion and absorption of fatty acids, the Peroxisome proliferator-activated receptor (PPAR) signaling pathway, and fatty acid degradation.

## 4. Discussion

Brown adipose tissue (BAT), as a specialized tissue dissipates energy into heat by non-shivering thermogenesis, displays morphological and functional plasticity in response to environment and pharmaceutical compounds stimulations. Recently, increasing number of bioactive compounds were reported to possess the capability to enhance brown adipogenesis. For instance, the activating effects of β3-adrenergic receptor agonists, rosiglitazone, corticosteroids, atrial natriuretic peptide, nitric oxide, as well as cold exposure on BAT differentiation and activation have been well-documented [18,24,25,26,27]. However, the potential crosstalk between these stimuli was often overlooked.

In mammals, acetate is the most abundant short chain fatty acid (SCFA) in the colon and systemic circulation. Notably, the health properties of acetate on body weight control and energy homeostasis have been extensively studied in the last decades [28]. Studies showed that acetate may prevent diet-induced body weight gain, counteracts adiposity, improves glucose homeostasis, and enhances insulin sensitivity [29]. However, there also exists evidence from animal studies showing that acetate promotes the development of obesity and insulin resistance [30]. Indeed, the controversies in the outcomes regarding to role of acetate in the metabolic health have drawn great attention, which has been referred to as ‘acetate discrepancies’ by some scholars [10].

Interestingly, these controversies were also found in studies regarding the role of acetate intervention on brown adipose tissue/brown adipocytes. For instance, abundant animal and limited human in vivo data suggest increasing the acetate availability via dietary sources may promote brown adipogenesis [31]. Several animal studies also highlighted the potential enhancing effects of oral acetate supplementation on energy expenditure via BAT [29]. Here, our study also suggested that that pre-treatment of acetate via drinking water may further enhance the effects of cold exposure on BAT, suggest that acetate intervention at the systemic level may promote the activation of BAT.

However, recently convincing evidence obtained from single-cell sequencing data suggested that the direct impact of acetate on brown adipocytes and brown adipose tissue is opposite. Local acetate administration at the interscapular brown adipose tissue even reduced the activity of the brown adipose tissue and caused its whitening [13]. Therefore, it is reasonable to presume the observation in our study that acetate administration via drinking water further enhanced the characteristic BAT gene upon cold exposure may mainly result from the overall impact of acetate on the whole body. Notably, it is plausible that acetate administration at the systemic level or gut microbially-derived acetate could affect host metabolism via a wide range of organs such as the central nervous system [32], liver [33], skeletal muscle [34], pancreas [35], and adipose tissue, and regulate a variety of physiological processes such as appetite regulation [36], lipid and glucose metabolism (e.g., glucose uptake, glycogen synthesis, lipogenesis and lipolysis, etc.) [37], insulin secretion [38], and adipokine secretion, etc. Indeed, as the important metabolites of anaerobic bacteria in gut, the acetate was also implied as a key regulator of intestinal homeostasis [39], intestinal barrier integrity [40], intestinal inflammation [41], etc. All these impacts could profoundly affect the body’s metabolism and thus influence the function of brown adipose tissue. Therefore, acetate intervention at the systemic level may exert different or even opposite functions compared to direct administration of acetate at specific organ. Indeed, a determination of local acetate level (e.g., at BAT) upon acetate intervention at the systemic level could provide useful information to understand its regulatory functions.

Notably, during the differentiation of preadipocytes into brown adipocytes, PPARs and C/EBPs are key activators of adipogenesis [5,42]. For instance, PPARγ could serve as a decisive factor in adipocyte differentiation [43] by regulating the expression of lipid metabolism-related genes and promoting the accumulation of triglycerides [7]. Meanwhile, C/EBPs regulate adipocyte differentiation by affecting glucose uptake and controlling the expression of lipogenic genes [6]. In addition, cAMP signaling networks (e.g., involving cAMP-dependent protein kinase, cAMP-responsive element-binding protein, etc.) also play crucial roles in stimulating adipogenesis and thermogenesis of brown adipocytes [44], while Foxo1 was also found to exert an important role in coupling insulin signaling to adipocyte differentiation [29]. Here, our GO analysis, KEGG pathway enrichment analysis, and GSEA analysis also highlighted that DEGs in BAT upon acetate treatment were closely related to these key pathways for brown adipocytes differentiation.

## 5. Conclusions

In summary, our study showed that acetate treatment via drinking water prior to a short period of cold exposure could induce obvious transcriptional changes in BAT. Particularly, the identified DEGs included a number of BAT-characteristic genes, suggesting there might be a potential synergistic effect between acetate pre-treatment and cold exposure regarding BAT activation. The underlying mechanisms may involve the upregulating signaling pathways governing adipogenic differentiation such as the cAMP signaling pathway, PPAR signaling pathway, etc. This finding may provide novel evidence for further understanding the roles of acetate intervention on BAT.

## Data Availability

The RNA-seq data in present study has been made publicly available to NCBI database (https://www.ncbi.nlm.nih.gov/) with the number PRJNA1005759, accessed on 15 August 2023.

## Supplementary Materials

The following supporting information can be downloaded at: https://www.mdpi.com/article/10.3390/biology12101285/s1, Figure S1: The transcriptional level of UCP-1 between mice with and without cold exposure.
