# Supplementary figures and images for: RNA-seq Analysis Reveals Potential Synergic Effects of Acetate and Cold Exposure on Interscapular Brown Adipose Tissue in Mice
Source: Biology (Basel). 2023 Sep 26;12(10):1285. doi: 10.3390/biology12101285 (PMC10603878; doi:10.3390/biology12101285)

Figure S1: The transcriptional level of UCP-1 between mice with and without cold exposure.

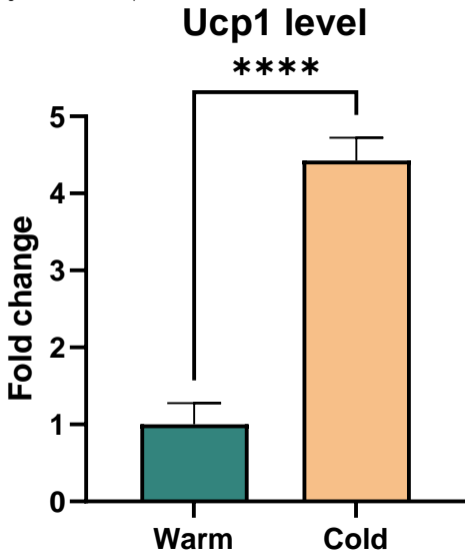

Supplement: Supplementary file 1 [file biology-12-01285-s001.zip › biology-2593581-supplementary.pdf]
